# Supplementary material for: Hierarchical Co(OH)2 Dendrite Enriched with Oxygen Vacancies for Promoted Electrocatalytic Oxygen Evolution Reaction
Source: Polymers (Basel). 2022 Apr 8;14(8):1510. doi: 10.3390/polym14081510 (PMC9026458; doi:10.3390/polym14081510)
Supplement: Supplementary file 1 [file polymers-14-01510-s001.zip › polymers-1629117-supplementary.pdf]

polymers

Article

Hierarchical $Co(OH)_2$ Dendrite Enriched with Oxygen Vacancies for Promoted Electrocatalytic Oxygen Evolution Reaction

Tingting Zhou [1], Zhen Cao [1,*], Xishi Tai [1,*], Lei Yu [1], Jian Ouyang [2], Yunfei Li [2] and Jitao Lu [1,*]

[1] College of Chemical Engineering and Environmental Chemistry, Weifang University, Weifang 261061, China; zhoutingting1986@163.com (T.Z.); jimiyulei@163.com (L.Y.)
[2] Shenzhen Kohodo Hydrogen Energy Co., Ltd., Shenzhen 518109, China; ouyangjian@kohodo.cn (J.O.); liyunfei@kohodo.cn (Y.L.)
* Correspondence: cczz250@163.com (Z.C.); taixs@wfu.edu.cn (X.T.); lujitao@foxmail.com (J.L.)

Citation: Zhou, T.; Cao, Z.; Tai, X.; Yu, L.; Ouyang, J.; Li, Y.; Lu, J. Hierarchical Co(OH)2 Dendrite Enriched with Oxygen Vacancies for Promoted Electrocatalytic Oxygen Evolution Reaction. *Polymers* **2022**, *14*, 1510. https://doi.org/10.3390/polym14081510

Academic Editors: Haolin Tang and Huacheng Zhang

Received: 22 February 2022
Accepted: 4 April 2022
Published: 8 April 2022

Publisher's Note: MDPI stays neutral with regard to jurisdictional claims in published maps and institutional affiliations.

Copyright: © 2022 by the authors. Submitted for possible open access publication under the terms and conditions of the Creative Commons Attribution (CC BY) license (https://creativecommons.org/licenses/by/4.0/).

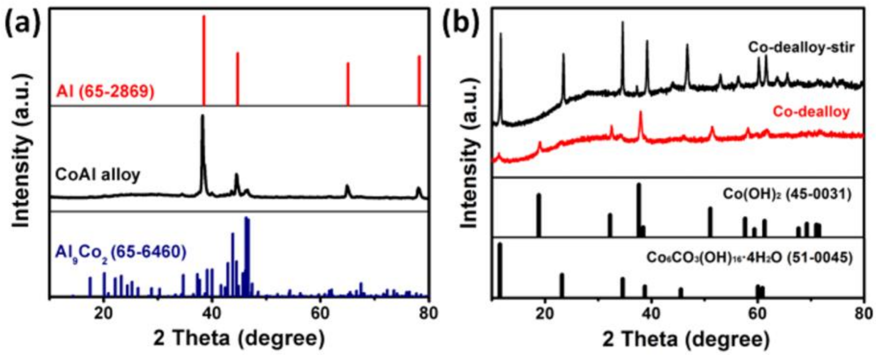

Figure S1. The X-ray diffraction patterns of (**a**) CoAl alloy ribbon precursor; (**b**) dealloying products with stir and without stir.

Considering stir could introduced more atmospheric carbon dioxide, the dealloying products with stir and without stir was investigated. The XRD patterns of the stir dealloying product almost entirely corresponding to $Co_6CO_3(OH)_{16}{\cdot}4H_2O$, which confirmed our hypothesis. Besides, as shown in Figure S2, the stir dealloying product ($Co_6CO_3(OH)_{16}$) possess the lower OER performance than the stasis dealloying product ($Co(OH)_2$). Considering the better OER performance and higher $Co(OH)_2$ content of stasis dealloying product, we employ stasis dealloying strategy to synthesize $Co(OH)_2$ and Ov-$Co(OH)_2$ NCAs in the following.

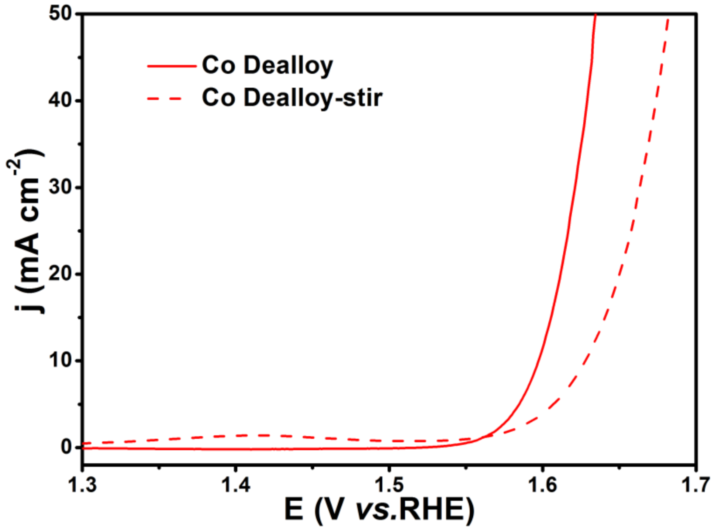

Figure S2. The Linear sweep voltammogram curves of dealloying products with stir and without stir.

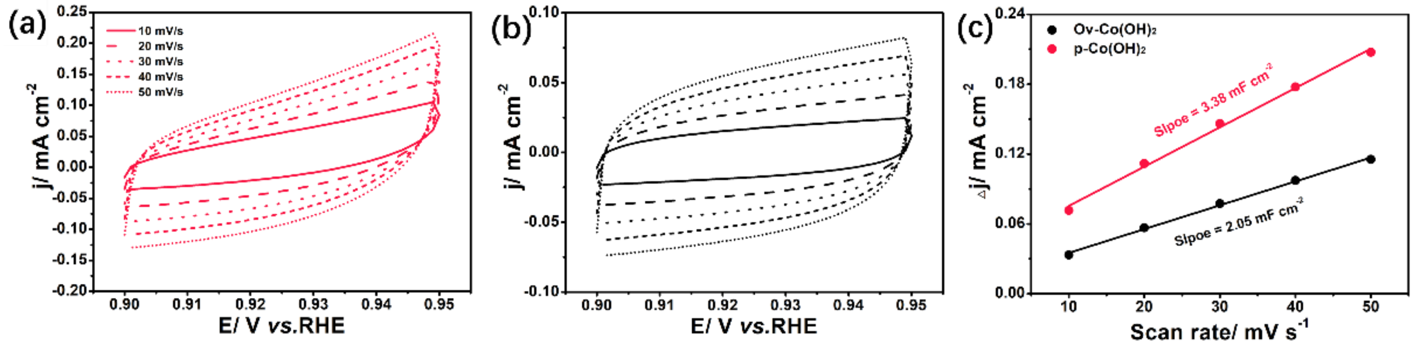

Figure S3. Cyclic voltammogram curves of (**a**) Ov-$Co(OH)_2$ and (**b**) *p*-$Co(OH)_2$ at the different scan rates from 10 to 50 mV s^{-1} in the potential range of 0.90–0.95 V versus RHE; (**c**) Capacitive currents at 0.93 V versus RHE as a function of scan rate for Ov-$Co(OH)_2$ and *p*-$Co(OH)_2$.

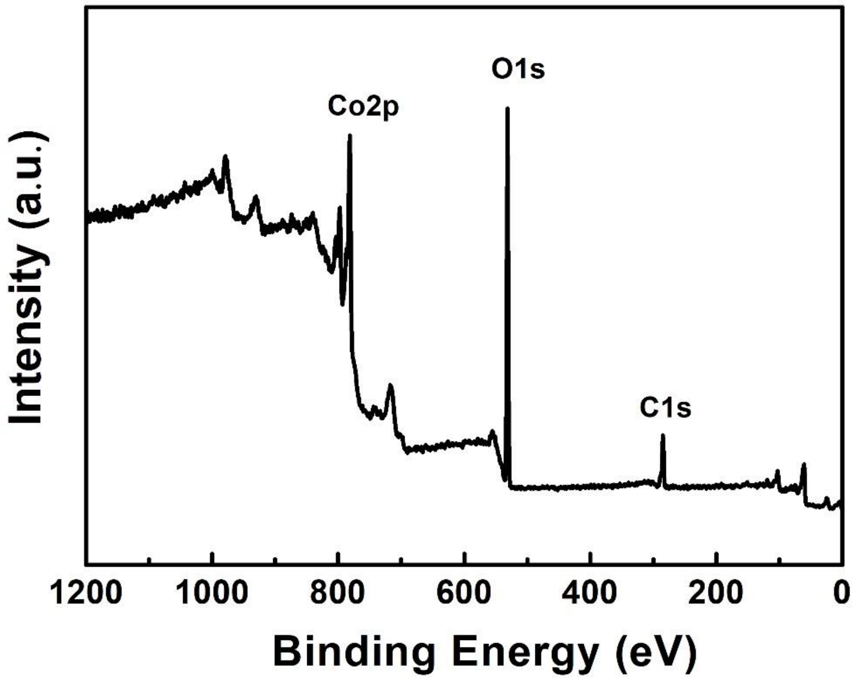

Figure S4. X-ray photoelectron spectroscopy survey spectra of *p*-$Co(OH)_2$.

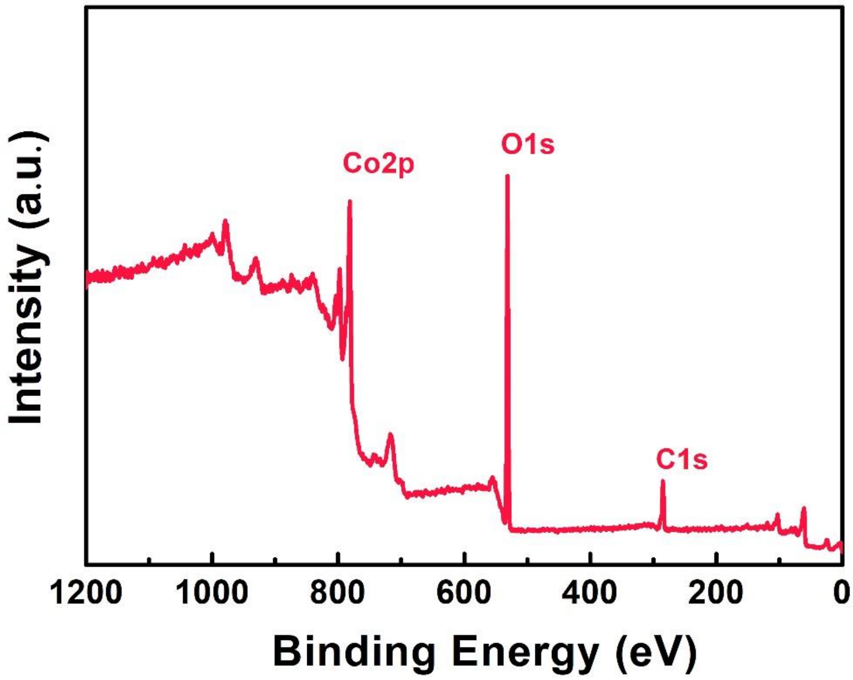

Figure S5. X-ray photoelectron spectroscopy survey spectra of O_V-$Co(OH)_2$.

In the XPS survey spectrum of O_V-$Co(OH)_2$ and *p*-$Co(OH)_2$, C, Co, and O are observed in both samples.

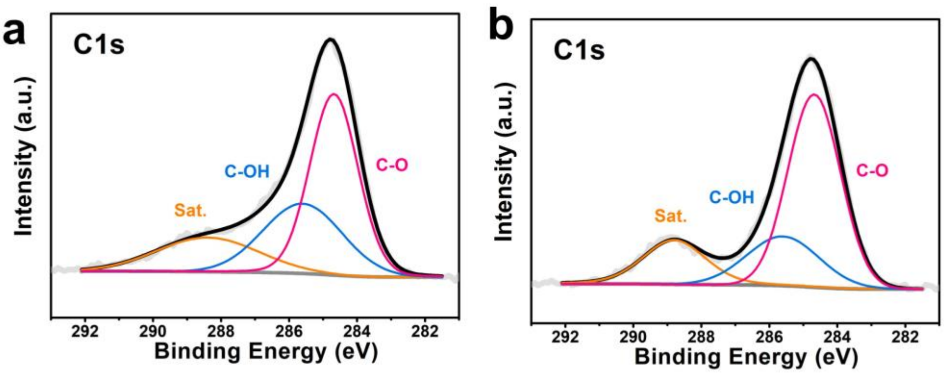

Figure S6. C 1s XPS spectra of (**a**) *p*-$Co(OH)_2$ and (**b**) Ov-$Co(OH)_2$.

Table S1. Atomic percentage of Al measured by XPS.

Sample	Atomic % of Al in Dealloy Products	Atomic % of Al in Alloy Foil	Removal % of Al
p-$Co(OH)_2$	3.39	95	99.2
Ov-$Co(OH)_2$	5.53	95	98.4
